# Goshajinkigan oxaliplatin neurotoxicity evaluation (GONE): a phase 2, multicenter, randomized, double-blind, placebo-controlled trial of goshajinkigan to prevent oxaliplatin-induced neuropathy

**DOI:** 10.1007/s00280-013-2306-7

**Published:** 2013-10-12

**Authors:** Toru Kono, Taishi Hata, Satoshi Morita, Yoshinori Munemoto, Takanori Matsui, Hiroshi Kojima, Hiroyoshi Takemoto, Mutsumi Fukunaga, Naoki Nagata, Mitsuo Shimada, Junichi Sakamoto, Hideyuki Mishima

**Affiliations:** 1 Advanced Surgery Center, Sapporo Higashi Tokushukai Hospital, Sapporo, 3-1, N33, E 14, Higashi-ku, Hokkaido, 065-0033 Japan; 2 Department of Gastroenterological Surgery, Osaka University, Graduate School of Medicine, Suita, Osaka, 565-0871 Japan; 3 Department of Biostatistics and Epidemiology, Yokohama City University Medical Center, Yokohama, Kanagawa, 232-0024 Japan; 4 Department of Surgery, Fukuiken Saiseikai Hospital, Wadanaka-cho, Fukui, 918-8503 Japan; 5 Department of Surgery, Aichi Cancer Center, Aichi Hospital, Okazaki, Aichi, 444-0011 Japan; 6 Department of Surgery, Sakai Municipal Hospital, Sakai, Osaka, 590-0064 Japan; 7 Department of Surgery, Kitakyushu General Hospital, Kitakyushu, Fukuoka, 800-0295 Japan; 8 Department of Surgery, Institute of Health Biosciences, The University of Tokushima Graduate School of Medicine, Tokushima, Tokushima, 770-8503 Japan; 9 Tokai Central Hospital, Kagamihara, Gifu, 504-8601 Japan; 10 Unit of Cancer Center, Aichi Medical University, Nagakute, Aichi, 480-1195 Japan

**Keywords:** Goshajinkigan, Peripheral neuropathy, Double-blind randomized trial, Oxaliplatin, Colorectal cancer

## Abstract

**Purpose:**

Oxaliplatin-induced peripheral neurotoxicity (OPN) is frequent and potentially severe, but successful treatment of this condition is still an unmet clinical need. We aimed to determine whether treatment with goshajinkigan (TJ-107), a traditional Japanese medicine, is better than placebo in preventing OPN in patients with advanced or recurrent colorectal cancer patients treated with standard FOLFOX regimens.

**Methods:**

In this phase 2, randomized, double-blind, placebo-controlled study, patients undergoing oxaliplatin-based chemotherapy were randomized to receive either oral TJ-107 (7.5 g) or matching placebo daily. The severity of OPN was assessed according to the Common Toxicity Criteria for Adverse Events at baseline, every 2 weeks until the 8th cycle, and every 4 weeks thereafter until the 26th week. The primary endpoint was the incidence of grade 2 or greater OPN until the 8th cycle of chemotherapy.

**Results:**

Analyses were done by intention to treat. Eighty-nine patients were randomly assigned to receive either TJ-107 (*n* = 44) or placebo (*n* = 45) between May 2009 and March 2010. The incidence of grade 2 or greater OPN until the 8th cycle was 39 and 51 % in the TJ-107 and placebo groups, respectively (relative risk (RR), 0.76; 95 % CI, 0.47–1.21). The incidence of grade 3 OPN was 7 % (TJ-107) vs. 13 % (placebo) (0.51, 0.14–1.92). No concerns regarding toxicity emerged with TJ-107 treatment.

**Conclusions:**

TJ-107 appears to have an acceptable safety margin and a promising effect in delaying the onset of grade 2 or greater OPN without impairing FOLFOX efficacy.

## Introduction

Oxaliplatin is considered one of the gold standard chemotherapeutic agents for advanced colorectal cancers and for adjuvant chemotherapy. However, oxaliplatin-induced peripheral neurotoxicity (OPN) is extremely common with the incidence varying from 82 to 98 % [1–3]. Severe OPN occurs in 10 to 20 % of patients [1, 4], and some may require dose reductions and discontinuation of treatment, potentially reducing the efficacy of chemotherapy and survival [5–7]. Despite considerable efforts to discover neuroprotective agents to prevent OPN [8], the best pharmacologic strategy for the management of OPN remains controversial [9–11].

In Japan, TJ-107 (goshajinkigan), a traditional Japanese medicine (Kampo) [12], has been frequently prescribed to alleviate symptoms of diabetic peripheral neuropathy such as numbness, cold sensation, and paresthesias/dysesthesias [13–15]. We hypothesized that TJ-107 might be effective against OPN and retrospectively investigated its use in a pilot study of 90 patients with advanced colorectal cancer undergoing FOLFOX therapy [16]. Patients were treated with TJ-107, calcium (Ca) gluconate and magnesium (Mg) sulfate infusion, combination of TJ-107 and Ca/Mg infusion, or chemotherapy alone. Our results showed that the group receiving TJ-107 with FOLFOX regimen experienced significant improvement in OPN and showed a favorable safety profile. We subsequently conducted a small, single-arm prospective study in 45 patients who were treated with modified FOLFOX6 for advanced colorectal cancer, in which 22 patients receiving oral TJ-107 reported lower incidence of grades 2 and 3 OPN than that in the control group [17]. Hence, this phase 2, randomized, double-blind, placebo-controlled, exploratory trial was initiated to investigate the neuroprotective effect of TJ-107 for OPN.

## Methods

### Study design

This was an exploratory, randomized, phase 2 trial to evaluate the efficacy of TJ-107 for preventing OPN in the Goshajinkigan Oxaliplatin Neurotoxicity Evaluation (GONE) study group conducted at 20 institutions in Japan [18].

### Eligibility criteria

Patients were eligible if they had histologically confirmed colorectal cancer and were scheduled to undergo chemotherapy with infusional 5-fluorouracil (5-FU), leucovorin (*I*-LV), and oxaliplatin (either FOLFOX4 or modified FOLFOX6 regimen). Patients had to have a good performance status (ECOG 0–1), adequate bone marrow function (WBC ≥ 3,000 and ≤12,000/mm^3^, neutrophil count ≥ 1,500/mm^3^, platelet count ≥100,000/mm^3^), renal function (serum creatinine level less than the institutional upper limit of normal), and hepatic function (bilirubin ≤ 1.5 times institutional normal, aspartate aminotransferase, and alanine aminotransferase levels less than 2.5 times the institutional upper limit of normal), life expectancy ≥12 weeks, without evidence of clinical infection, and without preexisting peripheral neuropathy from any cause. Exclusion criteria included prior exposure to chemotherapy, except for oral fluorinated pyrimidine derivatives or 5-FU/*l*-LV in an adjuvant setting, use of other Kampo medicines, history of severe hypersensitivity (allergy) to any medications, other active malignancies or a history of other malignancies within the past 5 years, congestive heart failure, diabetes, or a history of a hemorrhagic stroke. Patients who were pregnant or nursing, taking a neuropathic pain medication, or receiving radiation were deemed ineligible.

This study was conducted in accordance with the Declaration of Helsinki and the Japanese Ministry of Health, Labour and Welfare guidelines, and informed consent was obtained from all participants. The study protocol was approved by the local Institutional Review Board at each participating institution.

### Randomization and masking

Eligible patients were centrally randomized by a computer-generated allocation sequence in a 1:1 ratio to either TJ-107 group or placebo group. Information regarding the necessary follow-up tests was then sent to the registration center at the non-profit organization Epidemiological and Clinical Research Information Network (ECRIN). Patients, investigators, and data collectors were all blinded to treatment allocation.

### Study medications

TJ-107 is a mixture of aqueous extracts from 10 crude herbs in fixed proportions (Rehmanniae Radix, 10.7 %; Achyranthis Radix, 6.4 %; Corni Fructus, 6.4 %; Moutan Cortex, 6.4 %; Alismatis Rhizome, 6.4 %; Dioscoreae Rhizome, 6.4 %; Plantaginis Semen, 6.4 %; Poria (*Poria cocos* Wolf), 6.4 %; processed Aconiti Tuber, 2.1 %; and Cinnamomi Cortex, 2.1 %). The extract powder of TJ-107 is commercially available in Japan and was obtained from Tsumura & Co. (Tokyo, Japan). Matching placebo (Tsumura & Co.) was specifically manufactured for this clinical trial. The appearance, color, and odor of the placebo were well controlled that both patients and clinicians were unable to distinguish this placebo from the original TJ-107. Dried powder (2.5 g) of TJ-107 or placebo was administered orally three times a day before each meal (7.5 g/day).

### Treatment

Patients were randomly assigned to receive TJ-107 or placebo with either FOLFOX4 or mFOLFOX6 therapy. This treatment was initiated at the first delivery of FOLFOX and continued throughout the administration of chemotherapy and for 26 weeks beyond the completion of chemotherapy. Cycles of chemotherapy were given every 2 weeks until progressive disease or unacceptable toxicity occurred.

TJ-107 was given orally for 26 weeks starting on the day of oxaliplatin infusion. To avoid any possible influence on the assessment of neurotoxicity, Ca/Mg infusion was prohibited only during the 26-week administration of TJ-107 and not throughout the chemotherapy regimen. FOLFOX4 therapy consisted of infusion of *l*-LV at 100 mg/m^2^ over 2 h followed by 5-FU as a bolus (400 mg/m^2^) and a 22-h infusion of 5-FU (600 mg/m^2^) on day 1 and day 2, with infusion of oxaliplatin at 85 mg/m^2^ over 2 h on day 1. This regimen was repeated every 2 weeks. mFOLFOX6 therapy consisted of infusion of *l*-LV at 200 mg/m^2^ over 2 h followed by 5-FU as a bolus (400 mg/m^2^) and a 46-h infusion of 5-FU (2,400 mg/m^2^) with an infusion of oxaliplatin at 85 mg/m^2^ over 2 h on day 1. This regimen was repeated every 2 weeks.

Adverse reactions including OPN were assessed at baseline (prior to starting FOLFOX + TJ-107 or FOLFOX + placebo), every 2 weeks until the 8th cycle and every 4 weeks thereafter until the 26th week according to the National Cancer Institute Common Terminology Criteria for Adverse Events (NCI-CTCAE v3.0). The severity of neurotoxicity was assessed by the study clinicians who were blinded to treatment allocation and used the sensory neuropathy items in NCI-CTCAE v3.0, which describe the four grades as follows: grade 1, loss of deep tendon reflexes or paresthesia, including tingling, but not interfering with function; grade 2, objective sensory alteration or paresthesia, including tingling, interfering with function, but not interfering with activities of daily living (ADL); grade 3, sensory alteration or paresthesia interfering with ADL; and grade 4, permanent sensory losses that are disabling. In addition, patients rated their symptoms on a 0–4 scale using the Functional Assessment of Cancer Therapy/Gynecological Oncology Group-Neurotoxicity (FACT/GOG-Ntx-12) score at screening, at baseline, and before each chemotherapy treatment. These subjective ratings were independently evaluated from the clinician-rated CTCAE grading. The follow-up period was 1 year after registration of the last patient.

### Statistical analysis

The primary endpoint of this study was the incidence of grade 2 or greater OPN after 8 cycles of chemotherapy as assessed by the clinical investigators. The rate of incidence was compared between evaluable patients randomized to either the TJ-107 or the placebo group. The rate of occurrence of grade 2 or greater OPN was calculated for each group and compared using the chi-square test.

Secondary endpoints included the incidence and grading of OPN after each cycle, FACT/GOG-Ntx-12 score, time to occurrence of OPN, response rate to chemotherapy, and toxicity.

Previously, we found that the incidence of grade 2 or greater OPN was 15 and 45 % (TJ-107 vs placebo) from the start of oxaliplatin treatment until the completion of cycle 8 [16]. On the basis of this data, we determined that in order to detect with 80 % power while maintaining a significance level of 10 % in a two-sided test, 35 patients per group would be required to compare the two treatment groups with a chi-square test. To account for possible dropouts, a minimum of 40 patients were enrolled in each group (80 in total). Randomization was achieved by using three strata: use of bevacizumab, the institution, and the presence of target lesions evaluated by Response Evaluation Criteria in Solid Tumors (RECIST) version 1.1. This trial is registered with the UMIN Clinical Trials Registry in Japan (UMIN000002211).

## Results

**Fig. 1:**
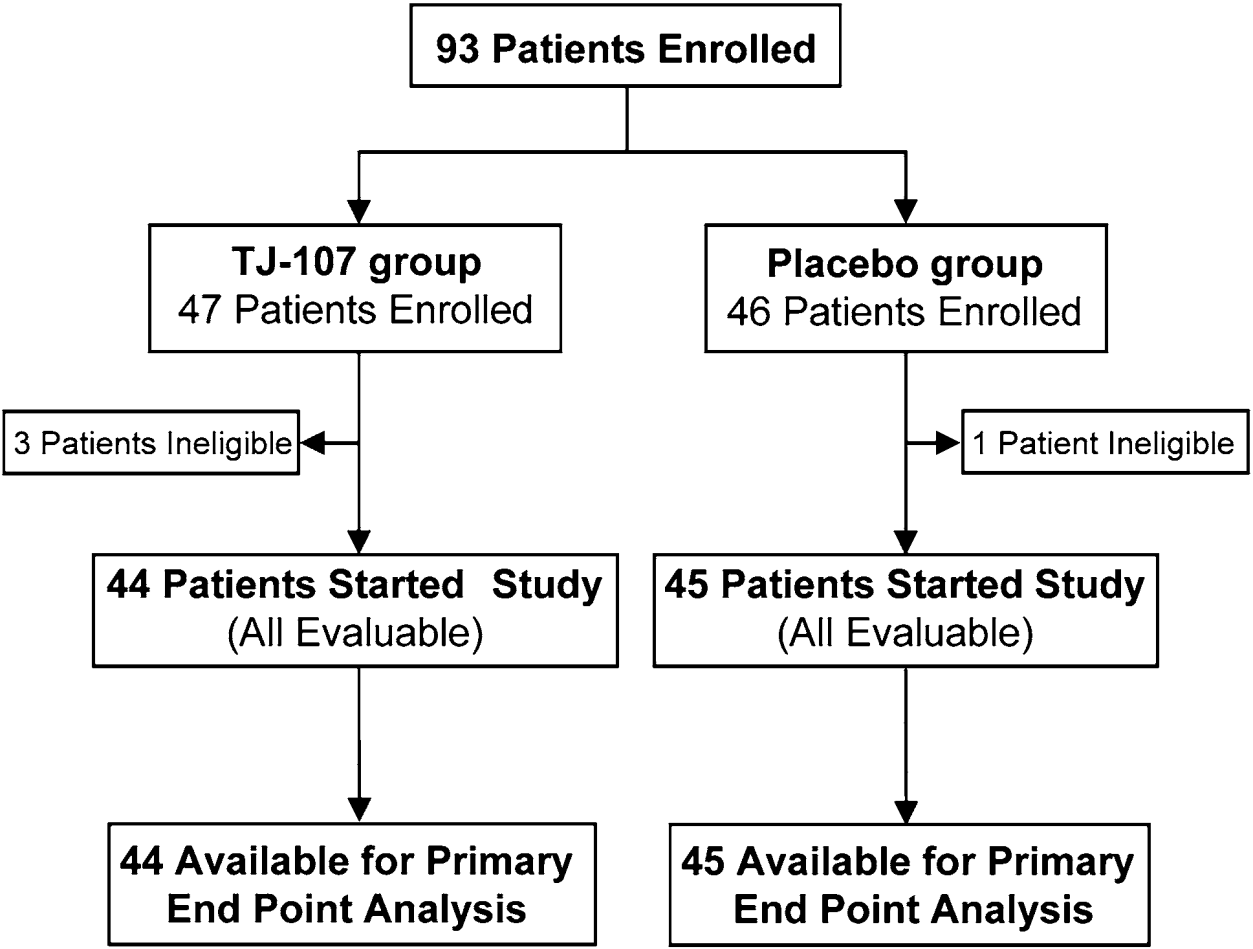
CONSORT diagram

A total of 93 patients were enrolled from May 1, 2009, to March 31, 2010. Of the 93 patients, 47 were assigned to the TJ-107 group and 46 to the placebo group. Four patients (3 receiving TJ-107 and 1 control) were withdrawn before initiation of treatment and included in the intention-to-treat set analysis (Fig. 1). The remaining 89 (96 %) patients were consequently assessable for efficacy and toxicity. Reasons for premature withdrawal were disease progression (6 patients), adverse events (4), medical reasons (4), patient request (2), and complete response, operation, or death (1 each). Generally, demographic and background characteristics of the patients were well balanced between the TJ-107 group (*n* = 44) and placebo group (*n* = 45) (*P* values ranging from 0.203–0.833) (Table 1).

**Table 1:** Patient characteristics

	TJ-107 (*N* = 44)	Placebo (*N* = 45)	*P* value
Sex			
Male	23	25	0.833
Female	21	20	
Age			
Median	67	61	0.215
Range	40–88	36–82	
Performance status			
0	40	44	0.203
1	4	1	
Primary tumor			
Colon	28	30	0.826
Rectum	16	15	
Chemotherapy			
First-line	36	35	0.793
Adjuvant	8	10	

### Incidence of OPN

Data on the incidence of grade 2 or greater OPN and grade 3 OPN until the 8th cycle are summarized (Table 2). The incidence of grade 2 or greater OPN until the 8th cycle was 39 % in the TJ-107 group and 51 % in the placebo group (relative risk (RR) 0.76; 95 % confidence interval (CI) 0.47–1.21). Similarly, the incidence of grade 3 OPN until the 8th cycle was 7 % in the TJ-107 group and 13 % in the placebo group (RR 0.51; 95 %CI 0.14–1.92).

**Table 2:** Oxaliplatin-induced peripheral neurotoxicity until the 8th cycle

Oxaliplatin-induced peripheral neurotoxicity until the 8th cycle			
	TJ-107 (*N* = 44) (%)	Placebo (*N* = 45) (%)	Relative risk [95 %CI]
Grade ≥2	39	51	0.76 [0.47–1.21]
Grade 3	7	13	0.51 [0.14–1.92]

**Fig. 2:**
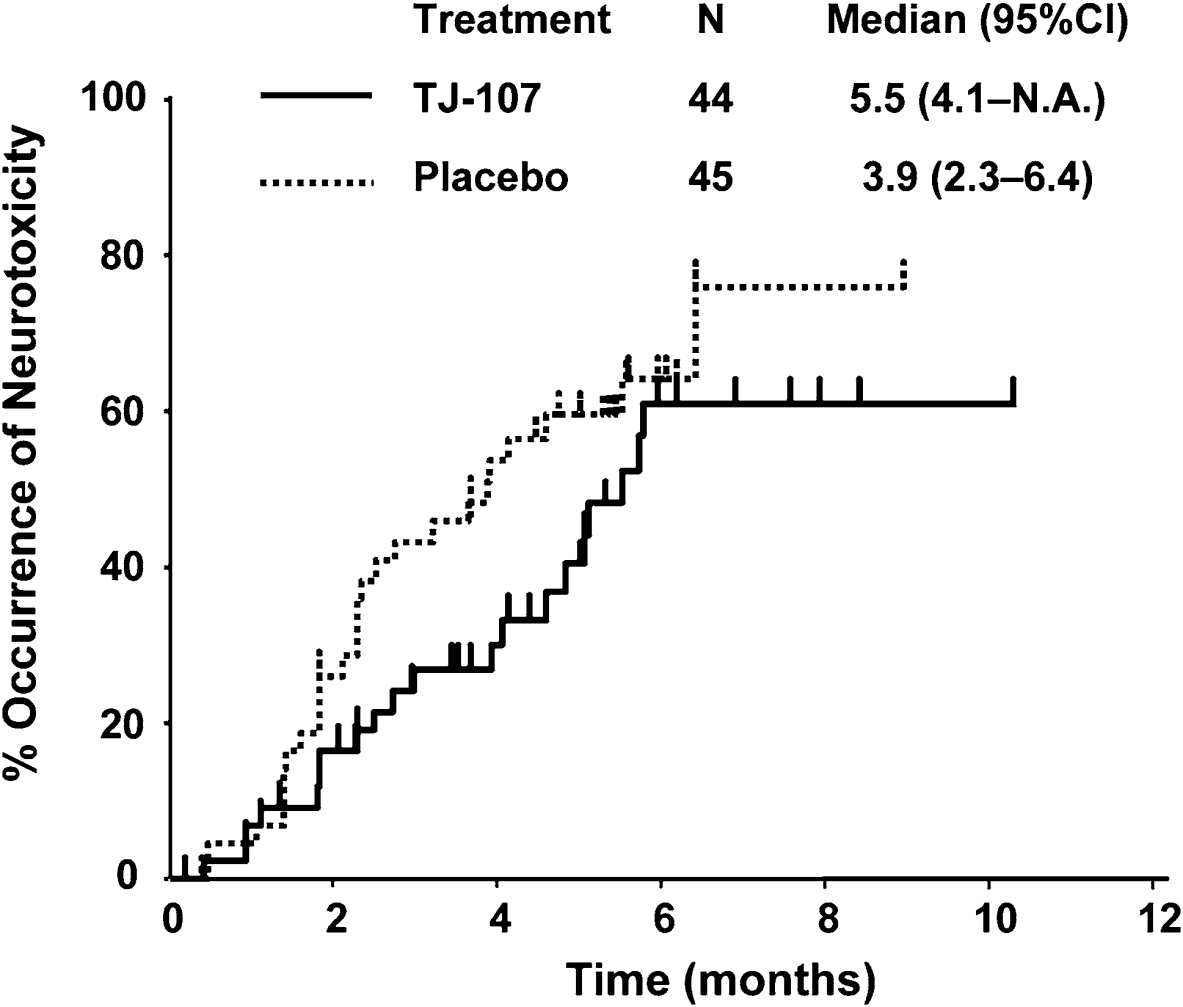
Time to occurrence of grade 2 or greater neurotoxicity. *Solid line* TJ-107, *broken line* Placebo

The time to occurrence of grade 2 or greater OPN is shown in Fig. 2. In patients who developed grade 2 or greater OPN, the median time to occurrence was 5.5 months (95 %CI 4.1–N.A.) in the TJ-107 group and 3.9 months (95 % CI 2.3–6.4) in the placebo group (RR 0.65; 95 % CI 0.36–1.17). The median time to occurrence of grade 3 neurotoxicity was better controlled in the TJ-107 group (RR 0.71; 95 % CI 0.29–1.77). The median frequency of occurrence of OPN at 26 weeks was 54.1 and 62.5 % (RR 0.86) in the TJ-107 and placebo groups, respectively.

When we stratified the patients based on FOLFOX regimen (i.e., FOLFOX4 vs. mFOLFOX6) and performed a subanalysis to determine whether there was a difference in the effects of TJ-107 on the occurrence of OPN, we found that there was no significant difference between treatment groups albeit the small sample size in each group.

The median FACT scores of the TJ-107 and placebo groups were 6.0 vs. 9.0 (*P* = 0.421) at 8 weeks and 7.0 vs. 10.5 (*P* = 0.151) at 26 weeks (Table 3). Although the differences were statistically unremarkable, patients receiving TJ-107 tended to show milder symptoms of neurotoxicity than those who received placebo.

### Tumor response rate

The anti-tumor effect was assessed in 27 (TJ-107) and 23 (placebo) patients who had a target lesion at the time of enrollment. Bevacizumab was administered in 74 % (20/27) and 74 % (17/23) of patients in the TJ-107 group and the placebo group, respectively (Table 4).

**Table 3:** Median FACT/GOG-Ntx-12 score

	TJ-107 (*N* = 44)	Placebo (*N* = 45)	*P* value
8 weeks	6.0	9.0	0.421
26 weeks	7.0	10.5	0.151

* FACT/GOG-Ntx-12: Functional Assessment of Cancer Therapy/Gynecological Oncology Group-Neurotoxicity-12 score

**Table 4:** Tumor response rate

Overall	TJ-107 (*N* = 27)	Placebo (*N* = 23)	*P* value
Complete response (CR)	1	1	
Partial response (PR)	14	10	
Stable disease (SD)	9	11	
Progression disease (PD)	3	1	
Not evaluable (NE)	0	0	
CP + PR 95 % CI	15 (56 %) 0.37–0.74	11 (48 %) 0.27–0.68	0.777
CR + PR + SD 95 % CI	24 (89 %) 0.77–1.00	22 (96 %) 0.87–1.00	0.614

The overall chemotherapy response rates were 56 % in the TJ-107 group and 48 % in the placebo group. In addition, 89 % (TJ-107) and 96 % (placebo) of patients demonstrated disease control (complete response, partial response, or stable disease) (Table 4).

### Toxicity assessment

TJ-107 used in this study appeared to be well tolerated. There were no significant differences between the two groups in terms of toxicity. The most common adverse events likely caused by the chemotherapy were anorexia, fatigue, nausea, and stomatitis, which were reported at similar rates from patients of both groups (Table 5). Vomiting was significantly suppressed in patients on TJ-107 compared with controls (9 vs. 29 %, *P* = 0.029). In the context of systemic chemotherapy, most of these events were likely related to chemotherapy-induced toxicity, yet none of them were considered TJ-107 related. Eighteen hematologic toxicity events of grade 3 or greater (15 neutropenia) in the placebo group were reported, and 12 events (10 neutropenia) were noted in the TJ-107 group (Table 6). One patient in the placebo group died as a direct result of progressive disease.

**Table 5:** Incidence of adverse events

All grades			
	TJ-107 (*N* = 44) (%)	Placebo (*N* = 45) (%)	*P* value
Fatigue	25 (57)	26 (58)	1.000
Anorexia	27 (61)	22 (49)	0.289
Nausea	20 (45)	28 (62)	0.139
Vomiting	4 (9)	13 (29)	0.029
Stomatitis	19 (43)	16 (36)	0.519
Diarrhea	15 (34)	10 (22)	0.245
Hand-food syndrome	11 (25)	7 (16)	0.302
Allergic reaction	8 (18)	4 (9)	0.230
Febrile neutropenia	0 (0)	2 (4)	0.494
Constipation	2 (5)	2 (4)	1.000
Ileus	0 (0)	2 (4)	0.494
Total Bilirubin	4 (9)	4 (9)	1.000
AST	13 (30)	23 (51)	0.052
ALT	10 (23)	19 (42)	0.070
ALP	13(30)	19(42)	0.271

**Table 6:** Incidence of hematologic toxicity events

	TJ-107 (*N* = 44) (%)	Placebo (*N* = 45) (%)	*P* value
All grades			
Leukopenia	21 (48)	27 (60)	0.291
Neutropenia	15 (34)	21 (47)	0.282
Anemia	30 (68)	31 (69)	1.000
Thrombocytopenia	12 (27)	15 (33)	0.646
Grade ≥ 3			
Leukopenia	1 (2)	2 (4)	1.000
Neutropenia	10 (23)	15 (33)	0.347
Anemia	1 (2)	1 (2)	1.000
Thrombocytopenia	0 (0)	0 (0)	1.000

## Discussion

This is the first placebo-controlled, randomized, exploratory study that assessed the efficacy of oral TJ-107 in the treatment of OPN in colorectal cancer patients undergoing oxaliplatin-based chemotherapy.

Our randomized, phase 2, exploratory trial using a placebo was designed to assess the potential success of oral TJ-107 in the phase 3 setting, rather than provide solid data on its efficacy. Results of this study showed promising relative risk that support the clinical activity of oral TJ-107 against OPN, particularly acute cold-associated neuropathy, in patients who received FOLFOX therapy (FOLFOX4 or mFOLFOX6) for colorectal cancer without imposing negative impact on oxaliplatin-based anti-tumor effect. Additionally, TJ-107 did not cause any adverse effects during the trial. Our findings, which showed improvement in both CTCAE grades and patient-rated FACT/GOG-Ntx scores, suggest that TJ-107 delays the occurrence of grade 2 or greater OPN during active treatment although its therapeutic effect may plateau after 6.5 months of continuous administration and that the development of neurotoxicity was not correlated with the completion of oxaliplatin treatment. Given the difficulty of generating statistically robust data with a small sample size, we surmised that our data warrant further investigation in a large phase 3 setting.

As an exploratory, phase 2 trial of patients with advanced or relapsed colorectal cancer (some with unresectable cancer), we conducted the endpoint assessment after 8 cycles rather than at treatment completion on the basis of a previous study that reported the high likelihood of detecting the side effects of oxaliplatin after 8 cycles [19], suggesting that this time point may be critical for deciding whether to continue oxaliplatin-based chemotherapy. Furthermore, a postmarketing drug surveillance of TJ-107 in Japan has shown that the median time to occurrence of grade 3 neuropathy with motor disorder was after 8 cycles. Taken together, these data suggest the evaluation of neuropathy at its peak incidence after 8 cycles to be clinically more meaningful than delaying the evaluation until treatment completion.

TJ-107 is a complex drug containing 10 medicinal herbs with a wide spectrum of pharmacologic actions [16]. Experimental studies have shown that TJ-107 relieves neurologic symptoms of diabetic peripheral neuropathy such as cold hyperalgesia and mechanical allodynia [13–15] primarily by the action of its analgesic component, detoxified Aconiti Tuber. The purported mechanisms by which this component works in concert with the other components of TJ-107 to exert a neuroprotective effect include (1) evoking the release of dynorphin and activating endogenous κ-opioid receptors to improve numbness or paresthesia [20, 21], (2) decreasing the release of transmitter proteins and sensory receptors associated with C-fiber nociceptor activation [22, 23], and (3) promoting nitric oxide production to improve blood supply to the nerves [24]. Furthermore, a recent experimental study has demonstrated that TJ-107 ameliorates the pain associated with OPN in rats without affecting the anti-tumor activity of oxaliplatin [25], which is in line with our findings. Interestingly, we also found that TJ-107 significantly decreased vomiting compared with placebo. The precise mechanism remains unclear but one of the components of TJ-107, Poria (*Poria cocos* Wolf), has shown an antiemetic effect through 5-HT3A receptor inhibition [26, 27].

According to the Multicenter International Study of Oxaliplatin, 5-Fluorouracil and Leucovorin in the Adjuvant Treatment of Colon Cancer (MOSAIC) study, the reported incidences of OPN were as follows: grade 1 (48 %), grade 2 (32 %), and grade 3 (12 %) [1]. In the present study, the incidence of grade 2 and grade 3 OPN in the placebo group until the 8th cycle was 51 % and 13 %, respectively, which is consistent with the MOSAIC study [1]. There was no marked difference in time to treatment failure in this study. One possible explanation for this is that even if greater than grade 2 OPN had occurred once, oxaliplatin administration was continued so long as OPN was downgraded to grade 2 on the day of administration. Another possibility is that patients may not acknowledge or report neuropathic symptoms for fear of missing out on an effective cancer treatment. Thus, it seems imperative to discover an agent that has sufficient evidence to decrease OPN development.

Many agents have been tested, in both humans and experimental animals, to ameliorate OPN [28]. Recently, the antidepressant drug venlafaxine, which is also used to manage pain associated with diabetic peripheral neuropathy, has been reported to significantly decrease the incidence of acute OPN in a placebo-controlled, randomized, phase 3 trial; however, grade 1–2 vomiting was observed more frequently in patients who received venlafaxine [29]. TJ-107 has also been used to treat painful diabetic peripheral neuropathy [13–15], but our study showed that TJ-107 significantly decreased vomiting compared to placebo, and other common adverse events due to chemotherapy did not worsen with TJ-107 treatment. Effective preventive treatments must not only mitigate neurotoxicity but must also preserve the antineoplastic effect of chemotherapeutic drugs. In this study, no between-group differences were found in response rates to chemotherapy or in the survival rates, suggesting that TJ-107 had no influence on FOLFOX therapy. Moreover, TJ-107 is an easily administered alternative that does not produce serious adverse effect, rendering it conducive to increasing compliance among patients and health care practitioners in a cancer treatment setting.

This is the first, randomized, phase 2, exploratory trial of TJ-107 whose study design itself is comparable to that of an older phase 3 trial design. The primary objective of this phase 2 trial study was to determine whether our findings would warrant validation in a phase 3 setting and not necessarily to obtain concrete data that show statistical significance. In other words, our aim was to define the characteristics of TJ-107 against OPN relative to placebo in order to refine the design of a phase 3 trial. Through this study, we were able to obtain a more accurate estimation of sample size and confirm that TJ-107 was similar to placebo in terms of toxicity despite the small sample size, and TJ-107 prevented the progression and development of severe neurotoxicity, one of the primary dose-limiting factors of oxaliplatin-based chemotherapy. Taken together, our findings suggest that this trial served as an effective platform for testing the efficacy of a novel agent like TJ-107 in oncology and for designing and accelerating the transition from phase 2 to a large phase 3 trial that employs objective measures.

## Conclusions

Findings from this phase 2, exploratory trial suggest that oral TJ-107 has acceptable margins of safety and tolerability and a promising effect in delaying the onset of grade 2 or greater OPN in colorectal cancer patients treated with oxaliplatin. 
